# China's Public Health Policies in Response to COVID-19: From an “Authoritarian” Perspective

**DOI:** 10.3389/fpubh.2021.756677

**Published:** 2021-12-15

**Authors:** Jinghua Gao, Pengfei Zhang

**Affiliations:** ^1^Centre for Social Investment (CSI), Max-Weber-Institute for Sociology, Heidelberg University, Heidelberg, Germany; ^2^School of Labor and Human Resources, Renmin University of China, Beijing, China

**Keywords:** public health, COVID-19, crisis management, authoritarian, health policies

## Abstract

**Background:** China is generally regarded internationally as an “authoritarian” state. Traditional definitions have assigned many negative connotations surrounding the term of authoritarian. We realize that it might not be considered value-neutral in other countries. But authoritarian in the Chinese context emphasizes more on centralized decision making, collectivism, coordinating all activities of the nation, and public support, which is considered a value-neutral term. Therefore, it is adopted in this paper. We would like to clarify this. Authoritarian governance is considered an important mechanism for developing China's economy and solving social problems. The COVID-19 crisis is no exception. Most of the current research on crisis management and government crises focuses on advanced, democratic countries. However, the consequences of crisis management by authoritarian governments have not been fully appreciated. Although prior research has addressed authoritarian initiatives to manage crises in China, authoritarian interventions have rarely been theorized in public health emergencies.

**Methods:** Based on a literature review and theoretical analysis, we use a descriptive and qualitative approach to assess public health policies and mechanisms from an authoritarian perspective in China. In light of the key events and intervention measures of China's government in response to COVID-19, the strategic practices of the Communist Party of China (CPC) to construct, embody, or set political goals through authoritarian intervention in public health crisis management are discussed.

**Results:** China's government responded to the COVID-19 pandemic with a comprehensive authoritarian intervention, notably by establishing a top-down leadership mechanism, implementing a resolute lockdown, rapidly establishing square cabin hospitals, enhancing cooperation between different government departments, mobilizing a wide range of volunteer resources, enforcing the use of health codes, imposing mandatory quarantine on those returning from abroad, and implementing city-wide nucleic acid testing. These measures ensured that China was able to contain the outbreak quickly and reflect on the unique role of the Chinese authoritarian system in responding to public health crises.

**Conclusions:** Our paper contributes to expanding the existing understanding of the relationship between crisis management and authoritarian system. China's response to COVID-19 exemplifies the unique strengths of authoritarian institutions in public health crisis management, which is a helpful and practical tool to further enhance the CPC's political legitimacy. As a socialist model of crisis management with Chinese characteristics, it may offer desirable experiences and lessons for other countries still ravaged by the epidemic.

## Introduction

Most countries in the world have been experiencing a serious public health crisis since the first case of COVID-19 infection was reported in Wuhan, China in December 2019. The impact of COVID-19 on China and the global economy is undoubtedly clear: It has caused a considerable number of infections and deaths in many nations and has become a global public health emergency (1). As the first country to respond, China's efforts—such as the implementation of lockdown measures and other preventive actions—appear to have been successful in containing the first wave of local transmission of the COVID-19 epidemic (2). Under the unified leadership and strategic deployment of the Communist Party of China (CPC) Central Committee and the State Council, China has achieved normal functioning of production and life and maintained sustained economic growth. While the situation in other countries has remained severe, China has been able to control large-scale transmission rapidly and has not continued to suffer from the serious impact of the epidemic, despite the emergence of confirmed cases in individual cities and regions.

Some studies have explored challenges (3), experiences (4), response strategies (5), and policy lessons (6, 7) for controlling COVID-19 and assessed China's public health system (8) and management mechanisms (9, 10). For example, China has used its national power to increase the number of doctors in areas experiencing severe pandemic situations to avoid a shortage of medical personnel (8). Similarly, China's experience in overcoming COVID-19 can be summarized as imposing an early and strict lockdown, implementing active case surveillance, rapid case diagnosis and effective contact tracing, and establishing temporary hospitals to accommodate the increasing influx of patients (4). However, such studies lack an “authoritarian” perspective in analyzing the reasons why China can control the epidemic so quickly.

China is generally regarded internationally as an authoritarian state (11–14). Authoritarian governance is considered an important mechanism for developing China's economy and solving social problems. The COVID-19 crisis is no exception: It represents a classic example of authoritarianism, with top-down centralization and close collaboration with local governments. Although previous studies have addressed authoritarian initiatives to manage crises in China, such as strong leadership and powerful interventions by the central government, authoritarian interventions have rarely been theorized in emergency public health crises. However, the existence of such phenomena and mechanisms reflect the policies of crisis governance and reveals the CPC's strategic practice in constructing, embodying, and setting political goals through authoritarian interventions.

In response to this gap, we review the practices and political logic of authoritarianism in the governance of public health crises in China, using the fight against COVID-19 as an example. We argue, first, that the governance practices of China's public health crisis contain some authoritarian elements, particularly centralization, coercive intervention, and state paternalism. Second, authoritarian intervention is both a useful and practical tool for responding to crises and an opportunity for the state to increase political legitimacy through crisis governance.

We first describe the development stages of COVID-19 in China. We then examine authoritarian initiatives in the governance of public health crises, particularly the lockdown of cities, the establishment of square cabin hospitals, nucleic acid testing, obligatory quarantine measures, and the mandatory use of health codes. Next, we discuss the relationship between authoritarianism and public health emergencies, the applicability of authoritarian crisis management in other countries, the relationship between the governance of COVID-19 and the CPC's political legitimacy, and how different political systems can be reconciled in international cooperation. Finally, we demonstrate how an analysis of authoritarianism in public health crisis governance in China extends the understanding of existing authoritarian systems.

## Literature Review

Traditional definitions have assigned many negative connotations surrounding the word authoritarian, such as “principle of blind submission to authority” and “a political system that concentrates power in the hands of a leader or a small elite” (15, 16). Authoritarian and democracy have become a dichotomous concept in the dominant discourse of Western political science. The authoritarian discourse tried to create the effect that those so-called authoritarian governments are always transitional, phased, and therefore unstable and unsustainable (17). We realize that this term might not be considered value-neutral in other countries, and even be entirely negative.

However, in the mid-1990s, some scholars argued that authoritarian governments possessed an advantage over more democratic governments in initiating and promoting economic growth, introducing the concept of authoritarian advantage (18). The idea of “soft authoritarianism,” as a general description of many Asian societies, especially for Singapore and China, has become an increasingly popular potential competitor to Western liberal democracy (19, 20).

Francis Fukuyama argues that soft authoritarian combines a market-oriented economic system with “a kind of paternalistic authoritarianism that persuades rather than coerces.” The resulting system is economically liberal, but politically quasi-authoritarian (21). In addition, soft authoritarianism emphasizes obedience to group interests rather than individual rights (19).

China is a country heavily influenced by Confucianism. Unlike Western democracies, authoritarian in Chinese context places more emphasis on centralized decision making, collectivism, coordinating of all activities of the nation, and public support (22, 23), which is considered a value-neutral term (24, 25).

A growing number of scholars have begun to use an authoritarian perspective to explain the social problems and phenomena in China. For example, the South-North Water Transfer Project (SNWTP) exemplifies the “authoritarian neo-liberalization” of China's water governance (26). Attention also has been paid to the innovative activities of authoritarian states in dealing with social crises (24). They concluded that critical crises are politically powerful and decisive in authoritarian systems (25). Nevertheless, in general, most of the current research on crisis management and government crises is focused on advanced, democratic countries (25). The consequences of crisis management by authoritarian systems have not been fully appreciated. Therefore, we choose to adopt the term of authoritarian in the paper.

Does authoritarian advantage exist in crisis management? Looking at China's performance in response to various crises, there seems to be a definite answer. The 2008 Sichuan earthquake brought much recognition to China's disaster relief policies. The Chinese government's unprecedented policy of information disclosure and its extensive cooperation with social and foreign organizations surprised observers both at home and abroad. In particular, the spurt in total social contributions and volunteer participation is often cited as a classic example of the government's ability to mobilize public support and increase the political legitimacy of authoritarian systems (27).

What is the situation in terms of the current public health crisis? Studies have found that authoritarian governments usually present themselves as more successful in controlling the spread of disease (28). Hofstede's cultural dimension of individualism vs. collectivism can serve as a powerful explanation for the difference in effectiveness of crisis response between Asian countries, which emphasize collectivism, and Western countries, which espouse individualism (29, 30). Because collectivist societies are supposed to cooperate more for the benefit of the majority, individual interests need to be sacrificed when necessary. Democracies, on the other hand, advocate for individual freedom, and governments must implement policies within the limits of what is legally permissible. Such institutional constraints inevitably cause numerous inconveniences in responding swiftly to disasters and crises.

Schwartz explored the advantages of authoritarian power in pandemic crisis management through a comparative case study of mainland China and Taiwan's responses to SARS. They argued that centralized decision-making power, public support for government initiatives, and the government's ability to shape the tone of crisis in the mass media led to the mainland's ultimately effective response to the epidemic (22). Innovative activities in response to social crises in authoritarian countries—such as the adoption of health codes, contactless service delivery, distance education delivery, public emotional comfort services, cross-border program promotion, cloud office adoption, and medical supply production—have all contributed to social crisis management. The case of China provides useful insights for other countries suffering from the COVID-19 crisis (24, 31).

However, under authoritarian systems, government transparency tends to be weakened, and the media is more easily controlled, resulting in the possibility that true numbers are underestimated. As a result, the steps taken by the Chinese government during the initial phase of the crisis did not show the advantages that an authoritarian political system should have (32). This has become the reason why the US accuses China of hiding real data. Some scholars have even hinted that China is deliberately manipulating information, deflecting responsibility, undermining trust in democracy, and underscoring the failure of democracy, thus propagating authoritarianism (33).

Despite the relatively strong ability of authoritarian states to exercise effective control over the internet and manipulate online opinion (34), China has not remained silent about the crisis. In the context of networked authoritarianism, social media can promote elements of both civic culture and institutional support (34). Authoritarian systems can reinforce their rule by allowing for open communication between citizens of a particular multiple opinion orientation (35). The case of China demonstrates how authoritarian system can adapt to the internet, and even use networked technologies to bolster legitimacy and strengthen their ability to govern society (36, 37). Moreover, civil society in one-party countries still shows strength and vitality in emergency services, funding, volunteerism, mutual aid, and materials in the face of the COVID-19 crisis (38).

It is clear from the results of the COVID-19 crisis response that China has been more successful in terms of confirmed cases and economic recovery than democracies such as the US and European states. Searching for the source of this mystery is noteworthy. After all, understanding and learning from the experiences of other countries is preferable to pointing fingers and shifting blame. Ending the ravages of this epidemic as soon as possible is consistent with the goal of safeguarding the health and well-being of all humanity, which is shared by all nations.

## Methods

We use a descriptive and qualitative approach to assess public health policies and mechanisms in China based on key events and interventions from an authoritarian perspective.

### Case Background

In December 2019, the first case of COVID-19 infection was reported in Wuhan. Widespread transmission occurred within a few weeks, with massive population movement during the Chinese Lunar New Year (39). Under the unified leadership and strategic deployment of the CPC Central Committee and the State Council, interventions implemented across China included the complete lockdown of cities, proactive case surveillance, rapid investments to improve detection capacity, the quarantining of cases, the treatment of severe cases, the quarantining of high-risk groups, and behavioral risk reduction strategies. These actions guaranteed that China would quickly contain the outbreak and prevent its widespread reemergence.

China's fight against the COVID-19 outbreak can be generally divided into five phases (40). Authoritarian interventions have been used throughout nearly the entirety of the pandemic response. In the first phase, Xi Jinping, general secretary of the CPC Central Committee, personally chaired a meeting of the Standing Committee of the Bureau of the CPC Central Committee, and issued instructions on the prevention and control of a possible epidemic of pneumonia of unknown cause in Wuhan. Premier Li Keqiang also hosted a meeting of the State Council to announce requirements for epidemic prevention and control. In addition, the National Health Commission set up a leading group for disease response, formed a national senior medical disease control team of experts, and sent a working group and an expert team to Wuhan to guide its response to the epidemic situation, and to conduct on-site investigations while issuing two versions of the “Diagnosis and Treatment Protocol for Novel Coronavirus Pneumonia.”

In the second phase, the Chinese government adopted a comprehensive authoritarian intervention in response to the pandemic. First, China's national leaders issued important instructions on the Wuhan epidemic, demanding that the safety and health of all people should be put first and at the top of the CPC's governing agenda. Second, resolute measures should be taken to lock down Wuhan, and to impose strict restrictions on people's mobility and exit routes from Hubei and Wuhan. Third, while sending a central working group, national resources were actively mobilized to support Hubei Province and Wuhan. This included dispatching national medical teams and organizing assistance from 19 other provinces to 16 cities in Hubei Province. Fourth, a Level 1 response (the highest level) for major public health emergencies was activated nationwide. Fifth, the timely release of information on the epidemic and the strengthening of international cooperation were called for. All party organizations and party members of the CPC had to remember the supremacy of the people's interests and the party's founding mission, and to lead the people in implementing the decisions of the CPC's central leadership.

In the third phase, the COVID-19 pandemic was largely controlled in China. President Xi Jinping reconvened a meeting of the Central Political Bureau and requested that differentiated control measures be taken for different regions to ensure the safety of the capital of Beijing with all efforts. With the national epidemic largely under control, it has become important to achieve coordination between epidemic prevention and socioeconomic growth, and to gradually resume normal work and daily life. To fulfill the CPC's commitment of building a moderately prosperous society by 2020, and to achieve the total elimination of poverty among the rural poor under existing standards, a statement was issued by President Xi that the adverse impact of the COVID-19 epidemic must be eliminated. This is a serious political commitment and political task for the entire country. Further, biosecurity was presented as part of China's national security.

In the fourth phase, China lifted outbound traffic restrictions in Wuhan and Hubei provinces, shifting the focus of prevention and control from preventing new cases on the mainland to preventing inbound cases. A series of strict measures was taken to prevent the entry of COVID-19 into the country and to stop its resurgence inside the country, such as the implementation of a 14-day mandatory quarantine observation and nucleic acid testing policy. At the same time, attention has been focused on the timely management of disseminated clusters of cases to prevent wider expansion of transmission.

In the fifth phase, epidemic prevention and control have become regular, ongoing tasks. China has stressed promoting economic growth. Local officials must accelerate the economy's high-quality transformation, achieve poverty eradication, and fully realize the goal of moderate prosperity. This includes stabilizing six fronts (employment, finance, foreign trade, inbound investments, domestic investments, and market expectations) and guaranteeing six priorities of jobs (daily living needs, food, energy, industrial and supply chains, the interests of market players, and the smooth functioning of grassroots government).

As of October 5th, 2021, 96,310 confirmed cases were reported in mainland China, with 838 existing confirmed cases (including 2 severe ones), 90,836 cumulative cured cases, 4,636 cumulative deaths, 1,203,454 cumulative close contacts traced, and 26,852 close contacts still under medical observation (41). In addition, as of September 18th, 2021, 217,404,043 doses of COVID-19 vaccine were reported, and the total number of vaccinated people reached 11,084,200 (of whom 102,207,000 were fully vaccinated), accounting for 78% of China's total population (42).

**Table:** The five stages of COVID-19 pandemic prevention and control in China.

**Stages**	**Name of Stage**	**Time Span**
Stage I	Swift response to the public health emergency	December 27th, 2019-January 19th, 2020
Stage II	Initial progress in containing the virus	January 20th–February 20th, 2020
Stage III	Newly confirmed domestic cases on the chinese mainland drop to single digits	February 21st–March 17th, 2020
Stage IV	Wuhan and Hubei: an initial victory in a critical battle	March 18th–April 28th, 2020
Stage V:	Ongoing prevention and control	Since April 29th, 2020

*Data source: The State Council Information Office of the People's Republic of China (PRC), www.scio.gov.cn*.

### Data Collection

Data sources in this paper include official websites of central and local governments, reports issued by the government, white papers, official statistics, academic journals, and media coverage articles.

### Official Statistical Data From the Chinese Government

Our official statistics are collected from the websites of relevant Chinese government departments, including the State Council of China (https://www.gov.cn), the State Council Information Office of China (http://www.scio.gov.cn/index.htm), the National Health Commission of China (NHC, http://www.nhc.gov.cn), and the Chinese Center for Disease Control and Prevention (CDC, https://www.chinacdc.cnNational).

### Government Documents About COVID-19

These key government documents we reviewed include those from the State Council, the National Health Commission, and the General Office of the State Council. These documents mainly refer to official studies, work circulars, prevention and control programs, management specifications, and guidance related to COVID-19, such as “Fighting COVID-19: China in Action,” as well as Government Work Reports, Guidance on the regularized prevention and control of CIVID-19 epidemic, Norms for the management of asymptomatic patients with novel coronavirus infection, and Solutions for the treatment of novel coronavirus pneumonia.

### Academic Journals and Media Articles

In addition, we analyzed Chinese academic and media documents to identify the Chinese government's discourse on authoritarian interventions, such as the CSSCI and SSCI journal articles, *People's Daily, Xinhua News Agency* (mouthpiece of CPC), *China daily* and *the Central Broadcasting Network*.

## Results

### Establishing a Top-Down Leadership Mechanism

Leadership, particularly during a pandemic, can be a lonely and difficult job (43). A better understanding of public leadership is critical for crisis response, especially in designing public health crisis policies (44, 45). China established an emergency management system with unified leadership, comprehensive coordination, classified management, hierarchical responsibility, and territorial management. When the epidemic spread from Wuhan to all of Hubei Province (and even other provinces), the CPC Central Committee immediately set up a leading group to respond to the epidemic. Other provinces quickly set up provincial COVID-19 prevention and control leading groups or working commands to be unified and responsible for the prevention and control of the epidemic in their administrative regions. A smooth and efficient leadership mechanism from the central to the local level helped the rapid spread of the epidemic to be controlled in a timely manner.

Apart from the top-down leadership groups from the central government to local levels, special meetings, speeches, and instructions from national leaders also play an irreplaceable role in the prevention and control of the COVID-19 epidemic. Reflecting on a behavioral orientation specific to leaders in Confucian-based culture (46), authoritative leadership seems to be closely linked to the Chinese political system. According to China's political tradition, any leadership collective must have a core; leadership without a core cannot be relied upon. China now requires all Communist Party members to obey the decisions and deployments of the Party Central Committee, with Comrade Xi Jinping as the core. The four consciousnesses of politics, general situation, core, and alignment must be firmly established. All Party members must unswervingly uphold the authority and centralized leadership of the Party Central Committee. President Xi held several meetings of the Standing Committee of the Political Bureau of the CPC Central Committee and personally directed the work of epidemic prevention and control, as well as the resumption of work and production. He stressed that:

*Party committees and governments at all levels and relevant departments should put the safety of people's lives and physical health in the first place, develop a thorough plan, organize all forces to carry out prevention and control, and take practical and effective measures to resolutely curb the momentum of the spread of the epidemic. We must make every effort to treat patients, identify the causes of virus infection and transmission as soon as possible, strengthen case monitoring, and standardize the disposal process. We should release information on the epidemic in a timely manner and deepen international cooperation. We also need to reinforce the guidance of public opinion, strengthen the propaganda and interpretation of pertinent policies and measures, and resolutely maintain the overall stability of society* (47).

The above statement reflects the special role of authoritative leadership in China's response to sudden public health crises; it highlights the focus and boosts confidence for the prevention and control of the epidemic. This is undoubtedly different from the governance systems of Western democracies. Under China's political system, statements by leaders can directly contribute to the introduction of legal policies that guide specific practical activities.

### Resolute Lockdown and the Rapid Establishment of Square Cabin Hospitals

Wuhan is a central city in the middle of China; it is strategically located and has convenient water, land, and air transportation. After December 2019, the outbreak of COVID-19 made Wuhan the focus of global attention. As China's 2020 Lunar New Year approached, cases imported from Hubei began to appear in multiple locations. The Chinese government imposed a lockdown on the population of Wuhan as well as all of Hubei Province (48). It lasted for 76 days—from January 23rd to April 8th, 2020—and not only contained the further spread of the epidemic in China's other provinces, but also bought the world valuable time to fight the epidemic, which may inform public health policy in other countries and regions (49).

After the lockdown of Wuhan, there was a spike in the number of patients seeking medical care. A large number of patients was moving around the community and society, causing a strain on medical resources. Hospital beds could not meet demand for infected cases. The central government made a choice, and required Wuhan to immediately and gradually transform a number of sports stadiums and convention centers, and to adopt large-scale square cabin hospitals to prevent and control the epidemic. A portable medical space is a major initiative in China's public health prevention and control, allowing for both rapid alteration and swift recovery, and enabling high speed, low costs, and high efficiency in controlling the source of infection and treating patients.

### Cooperation Mechanism Between Different Government Departments

The prevention and control of the COVID-19 outbreak involves multiple government agencies (10). Different departments have different responsibilities in emergency management and need to work with each other. The Ministry of Emergency Management, as China's emergency response department, oversees primary management and overall coordination. Provincial and municipal health planning commissions play a professional role in the emergency management of public health emergencies as specialized departments in the field of health. Other relevant departments assist in the prevention and control of the outbreak in their respective areas. Specifically, the transportation department implemented traffic control to contain the spread of the epidemic. Human resources and social security departments should safeguard the legitimate rights and interests of employees and ensure the stability of labor relations. Financial departments need to allocate funds to support local governments in carrying out basic public health services and epidemic prevention and control at the community level. The Department of Health Insurance expands the coverage of medical insurance and exempts patients from the burden of health care. The Ministry of Education is in charge of introducing policies to adjust or delay the start of local primary and secondary schools and kindergartens. Radio and television departments should step up publicity on the epidemic to boost the public's confidence in fighting the epidemic. For example, China's Henan province—which borders the worst-hit province of Hubei—launched a joint prevention and control mechanism at the beginning of the epidemic, requiring 31 government departments to share information and form a joint prevention and control system (9). In addition to domestic efforts, the most notable progress in policy engagement to control early outbreaks has been achieved through transparent and trusting collaboration with global health practitioners (10).

### Establishing the Interprovincial Assistance Mechanism

As a kind of social risk, public health emergencies are often not limited by region and time. Regional risk avoidance paths focusing only on some groups and local areas easily fail (50). This is an important reason for the emergence of aid mechanisms (51). Contagious public health events require coordination and communication among local governments. China has learned to respond to emergencies in the sense that whenever a party is facing difficulty, the entire society musters support and comes together to cope with challenges. Wuhan and Hubei Province, as the worst affected areas, received strong support from other provinces. In accordance with the unified deployment of the CPC's Central Committee and under the coordination of the National Health Commission, 19 provinces sent medical teams to support 16 cities, states, and counties in Hubei Province in such a way that one province was responsible for one city. A total of 42,000 medical personnel from all over the country have worked side by side with local medical personnel in Hubei, making important strides in the prevention and control of the epidemic (52). This is a great feat in the history of fighting the epidemic and has won China high recognition and widespread praise from the international community.

### The Extensive Mobilization of Social Forces

In modern society, only the establishment of cooperative governance mode of multiple subjects—including governments at all levels, professional institutions, and social organizations—can effectively resolve risks (53). Since the outbreak of COVID-19, the Chinese government has actively mobilized various enterprises, public organizations, volunteers, social workers, and other social forces to participate in the emergency response system. The financial, material, and voluntary support provided to the fight against the epidemic has highlighted the non-profit sector's critical role in the emergency response system. A large number of social workers and volunteers took the initiative to participate in the front-line work of epidemic prevention and control, providing psychological counseling, humanistic care, and relationship adjustment services for confirmed cases, and solving the problems of travel, food, and the distribution of daily supplies for many front-line medical personnel. Socially responsible companies and individuals at home and abroad took the initiative to help increase the availability of protective clothing, masks, and other materials to assist in preventing and controlling the epidemic. The extensive participation of social forces has provided an important supplement to funds for fighting the epidemic. Further, the government actively mobilized enterprises and non-profit organizations to support the construction of medical and health disciplines, personnel training, and medical research and development.

### The Mandatory Adoption of the Health Code

The “Epidemic Prevention Health Information Code” in China is a nationally recommended, interoperable, and mutually recognized electronic certificate of personal health information. Generally, health codes need to be presented when entering office buildings, shopping malls, subways, train stations, and other locations with a high pedestrian flow. Using the color of the health code, an individual can be quickly identified whether the pathway is through the provinces and cities, where the epidemic is more serious, and whether he/she has been in direct or indirect contact with infected cases. Then, through big data analysis, it is possible to target directly and find people who may be infected. In other words, the use of health codes can dynamically monitor the health of each person, simplify the manual registration process, and enhance the accuracy of epidemic prevention and control. However, elderly people face some obstacles in using smart technology. It is difficult for them to learn to operate smart devices, to input data on their cell phones, and to open health codes when they take public transit and go to shopping malls and supermarkets. This has led to them becoming a uniquely disadvantaged group in the digital economy. The issue of aging and the use of smart devices by the elderly have become a real problem for China's society, and is also an important issue of great public concern.

### Forced Quarantine for Those Returning From Abroad

Since the COVID-19 epidemic is still spreading in overseas countries, the situation is relatively serious. Some overseas students and workers are more willing to return to their home countries. China's General Administration of Customs fully launched a health declaration system in March 2020, requiring all incoming personnel to make health declarations. In terms of quarantine measures, China conducted temperature monitoring screenings and medical inspections for both Chinese and foreign citizens entering the country. For those found to have symptoms from countries or regions where the epidemic is more serious, or those who have been in contact with confirmed or suspected cases, customs will strictly implement epidemiological screening, medical screening, and laboratory testing for screening. Four categories, confirmed cases, suspected cases, symptomatic persons, and close contacts need to be transferred, quarantined, and detained in accordance with the relevant regulations. The purpose is to achieve early detection, timely reporting, prompt quarantine, diagnosis, and treatment (54). On the basis of the 14-day quarantine medical observation measures for inbound personnel, they are required to strictly implement home health monitoring within 7 days after release from quarantine, to do a good job of personal protection when going out, and to avoid participating in activities where people tend to congregate. Nucleic acid testing was still required once on the next day and once on the 7th day after being released from quarantine. If symptoms such as fever and cough appear, it is necessary to seek medical attention in a timely manner to reduce the risk of transmission effectively when individual entrants are positive for nucleic acids after release from quarantine (54, 55).

**Table:** Entry quarantine and health testing regulations in certain Chinese provinces.

**Cities/Provinces**	**Quarantine measures**	**Number of nucleic acid tests**
Beijing	14 days of centralized quarantine + 7 days of home or centralized quarantine + 7 days of health monitoring	Several
Shanghai	14 days of centralized quarantine + 7 days of home health testing	6
Wuhan	14 days of centralized quarantine + 7 days of home isolation + 14 days of community health management	Several
Hong Kong	21 days of mandatory quarantine	several
Guangzhou	14 days of centralized quarantine + 7 days of home isolation	8
Shenzhen	14 days of centralized quarantine + 7 days of home health monitoring	2
Nanjing	14 days of centralized quarantine + 14 days of home isolation	2
Tianjin	14 days of centralized quarantine + 7 days of home isolation	3
Zhengzhou	14 days of centralized quarantine + 7 days of home medical observation	1 nucleic acid + 1 serum antibody test
Guangdong Province (Except Shenzhen and Guangzhou)	14 days of centralized quarantine + 7 days of home isolation	4
Anhui Province	14 days of centralized quarantine + 7 days of home isolation + 7 days of health monitoring	3
Zhejiang Province	14 days of centralized quarantine medical observation + 7 days of home health observation + 7 days of daily health monitoring	5
Liaoning Province	14 days of centralized quarantine + 7 days of home isolation (centralized quarantine if not eligible for home isolation) + 7 days of community health monitoring	4 times nucleic acid + 1 serum antibody test
Hunan Province	14 days centralized quarantine (first point of entry) + 7 days centralized quarantine (place of residence) + 7 days home health monitoring	3
Jiangxi Province	21 days of centralized quarantine + 7 days of home medical observation	5

*Source: Collected according to local epidemic prevention and control regulations*.

### City-Wide Nucleic Acid Testing

During the normalization stage of epidemic prevention and control, the COVID-19 epidemic in China came to be characterized by multiple points of occurrence and local outbreaks. City-wide nucleic acid testing has been carried out in some cities and regions one after another, which is one of the most important means of rapidly identifying infected cases so that they can be isolated, effectively cutting off the transmission route and preventing the spread of the virus. The goal is to contain the outbreak to a minimum as quickly as possible. Thus, the National Health Commission of China issued a notice to strengthen the organization and management of whole-person nucleic acid testing in August 2021. The notice puts forward the following requirements. For example, localities should formulate and improve the implementation plan for city-wide nucleic acid testing, enhance organizational coordination, strictly regulate the reporting of positive cases, and reinforce information technology support. The notice also underscores that once the implementation of city-wide nucleic acid testing is determined, it should be ensured that 5 million people at minimum complete the test within 2 days; documentation for a number of people >5 million must be completed within 3 days (56). City-wide nucleic acid testing has been conducted in several cities such as Nanjing, Wuhan, Guangzhou, Dalian, Zhengzhou, Shijiazhuang, Qingdao, and Heilongjiang Province.

## Discussion

First, we need to discuss the relationship between authoritarian systems and crisis management. Some scholars argue that there is no single political system best suited to crisis management as different systems have distinctive strengths and weaknesses, and any advantages of the system are largely offset by key drawbacks (32). However, it seems that for many authoritarian states like China, government intervention is much easier, and they have been able to put in place effective containment policies with ease (57).

China's COVID-19 crisis management provides the latest evidence of authoritarian institutional interventions in public health crisis prevention and response. In the early stages of the epidemic, the Chinese government's decision to lock down Wuhan, establish square cabin hospitals, and implement centralized quarantine of infected cases all reflect the resolute measure of the authoritarian system in the face of emergencies. During the response phase of the epidemic, the central government's initiatives reflect how China launched an unprecedented national campaign to contain the pandemic (58). In particular, the establishment of a top-down leadership mechanism (59, 60), interprovincial assistance (61, 62), and the mobilization of volunteer resources (63) are all valuable experiences in dealing with public health emergencies in China. Although hostility toward China is still widespread in many parts of the world, clearly that China has handled this unprecedented public health crisis fairly quickly since the lockdown of Wuhan (64).

Therefore, China's case powerfully demonstrates how authoritarians used the COVID-19 crisis to consolidate the government's legitimacy (65). The legitimacy of the CPC's rule is based on the hearts and minds of the people. Only when it is supported by the people is it the most concentrated expression of whether the ruling party's line and policies are in accordance with the fundamental interests of the greatest number of people. It also constitutes the fundamental measure of a society's political identity and the basic principle of governing legitimacy. COVID-19 took place during a critical time when China was building a moderately prosperous society and winning the battle against absolute poverty, which provided both an opportunity and a challenge for the rulers to demonstrate or extend their legitimacy by responding to the crisis. Only by winning this battle can people's hearts and minds be won, and the authoritarian system's legitimacy be consolidated. This is definitely the fundamental reason and driving force behind the Chinese government's rapid control of the COVID-19 outbreak.

Do other authoritarian countries also have advantages in responding to the COVID-19 epidemic? It does not appear to be true. By analyzing the performance of Iran and Turkey in dealing with the COVID-19 crisis, some scholars argue that Turkey did a “better” job than Iran in responding to the COVID-19 public health crisis (28). Even though most authoritarian governments are more likely to institute strict censorship of major media, they try to control the flow of news and information to the public (66). Typically, authoritarian systems' media play a stronger role in maintaining the stability of the system than in liberal systems (67). Only some authoritarian systems are good at taking forceful action. For example, both China and Russia inhibited crucial epidemic information, but only China chose and implemented truly effective actions (68). Therefore, we cannot say that there is a real authoritarian advantage in crisis management. Due to differences in government systems and healthcare infrastructure, in a decentralized system of government, it is unrealistic to try to replicate the Chinese model of crisis management (69).

Even in China, the early reaction of local officials to the outbreak fits a familiar pattern of bureaucratic behavior from its past, which was to make problems go away at all costs (70). Normally, localized governments tend to give insufficient or excessive warnings when dealing with public health emergencies. Under pressure to assess the performance of local officials, some local government leaders are reluctant to announce early-warning information during a public health crisis. This runs counter to China's human-centered early warning systems and could ultimately lead to a catastrophe (71).

Further, the ongoing COVID-19 pandemic has heightened discussion of the use of mobile phone data during the response to the outbreak (72, 73). Government departments in China have access to, and use, personal smartphone application data to track the activities of individuals. The use of cell phone data raises legitimate public concerns about privacy, data protection, and civil liberties (74, 75). Measures such as strictly controlling the mobility of the population, providing proof of negative COVID-19 nucleic acid tests, and showing health codes in public places have caused strong resentment and non-compliance. For older groups without smartphones, these steps seem less friendly, sparking discussions of inequality and the digital divide (75). It not only affects their normal lives, but also leads to the inability to participate in society actively. In the future crisis management of public health emergencies, there is a lot of room for improvement in terms of how to make reasonable assessments, scientific warnings, and the fair application of digital technologies.

Finally, we discuss how different polities can be reconciled in the context of international cooperation in the face of global crises. COVID-19 highlights governments' limited capacity to govern in critical areas. The emergence of pandemic diseases reflects the complexity of global ecosystems (76). Urbanization, globalization, destruction of biodiversity and inappropriate meat consumption are exacerbating the socioeconomic and environmental drivers of the epidemic (77). This leads to increased human vulnerability to disasters and hinders an effective disaster response. Due to expanded worldwide mobility and interconnectedness, regional epidemics are more likely to develop into pandemics (78, 79). China's pandemic management needs to consider integration into the larger global landscape or prevention of emerging infectious disease risks. In future international crisis governance, governments at the national level will need to strengthen international cooperation (80). It is undeniable that reconciliation of different polities in response to global public health crises is a huge dilemma. The crisis also exposed the failure of the international order to respond to the epidemic. Instead of acting collectively to save the world, the superpowers fell into a trap of meaningless competition and wasted efforts fighting the virus (65). The sensible approach is to abandon inter-institutional stereotypes and to prioritize social justice and equity for effective disaster management and risk reduction. Based on this, the world urgently needs to set up an international framework for cooperation that transcends different systems (81, 82), within which global efforts can be coordinated to address future global health. As long as it is conducive to eliminating the global public health crisis as soon as possible and maximizing the protection of people's lives and health, it is better to build understanding of, and respect for, each other's choices, and to collaborate with them (83).

## Conclusion

The COVID-19 crisis is the most serious public health crisis that has occurred since the foundation of the PRC. In contrast to existing studies on crisis management of public health emergencies, we explain authoritarian interventions in China's crisis governance by reviewing the response practices of COVID-19. Through a case study of COVID-19 governance, our paper contributes to expanding the existing understanding of the relationship between crisis management and authoritarianism. The contradictory and selective adaptation of authoritarianism in the handling of public health crises in China shows disadvantages in the discovery phase of an epidemic, and advantages in responding to a pandemic.

Our findings suggest that China's COVID-19 pandemic response included some essential elements of authoritarianism, particularly firm leadership, strong government intervention, and the implementation of authoritarian measures. This includes direct intervention by national leaders in crisis management, the decisive lockdown of Wuhan, the establishment of cross-provincial cooperation mechanisms, city-wide nucleic acid testing, mandatory quarantine policy and use of health codes. China's response to COVID-19 reflects the unique strengths of authoritarian institutions in public health crises. Crisis management—as a way to win the hearts and minds of the people—has become a source of political legitimacy for the CPC. It is a helpful and practical tool to further enhance its political legitimacy. As a socialist model of crisis management with China's characteristics, it may offer some desirable experiences and lessons for other countries still ravaged by the epidemic.

## Data Availability Statement

The original contributions presented in the study are included in the article/supplementary material, further inquiries can be directed to the corresponding author/s.

## Author Contributions

JG conceptualized, designed the study, and performed the analysis. JG and PZ wrote the first draft. PZ supervised the research. All authors have read and agreed to the published version of the manuscript.

## Conflict of Interest

The authors declare that the research was conducted in the absence of any commercial or financial relationships that could be construed as a potential conflict of interest.

## Publisher's Note

All claims expressed in this article are solely those of the authors and do not necessarily represent those of their affiliated organizations, or those of the publisher, the editors and the reviewers. Any product that may be evaluated in this article, or claim that may be made by its manufacturer, is not guaranteed or endorsed by the publisher.
